# Postpartum-Onset Moyamoya Disease: A Rare Cause of Stroke in Unexpected

**DOI:** 10.1155/2020/7689450

**Published:** 2020-07-15

**Authors:** Muhammet Ozer, Khadija Merchant, Zulfiya Manning, Suleyman Yasin Goksu, Kirti Juneja, Vernard S. Fennell

**Affiliations:** ^1^Department of Internal Medicine, Capital Health Regional Medical Center, Trenton, NJ, USA; ^2^Department of Internal Medicine, University of Texas Southwestern Medical School, Dallas, TX, USA; ^3^Bharati Vidyapeeth Deemed University Medical College, Pune, Maharashtra, India; ^4^Division of Endovascular Neurosurgery, Capital Health Regional Medical Center, Trenton, NJ, USA

## Abstract

Moyamoya disease (MMD) is a chronic cerebrovascular occlusive disease that is characterized by progressive bilateral stenosis of the terminal portion of the internal carotid artery and its main branches. Cerebrovascular events are the primary presenting symptoms and are related both to stenosis and occlusion of the ICAs and their main branches. Detection of bilateral stenosis by cerebral angiography is considered the gold standard, but computed tomography angiography (CTA) is also an acceptable method of diagnosis. In the current literature, there are no precise data on the incidence of moyamoya disease in Europe and the United States. Also, the pathogenesis of MMD remains obscure, and genetic factors and inflammation are the two most representative mechanisms. Here, we report the case of MMD in a 29-year-old African American female who presented with an ischemic stroke for the second time that manifested after pregnancy. This case is important to increase awareness of the probability of this rare disease in Western countries as well as to call attention to pregnancy's accelerating effects of MMD. Careful, long-term neurologic and radiologic follow-up is essential in adult patients with MMD to prevent additional stroke events and improve outcomes.

## 1. Introduction

Moyamoya disease (MMD) is a chronic progressive cerebrovascular occlusive disease characterized by bilateral stenosis of the terminal portion of the internal carotid artery and its main branches accompanied by an abnormal vascular formation of collateral vessels at the base of the brain. Significant presenting symptoms of MMD are transient ischemic attacks or strokes. Takeuchi, for the first time, described MMD in 1957 in Japan [1]. Moyamoya means “a puff of smoke” in Japanese. The reason why the name was given is due to the typical appearance of collateral blood vessels that develop at the base of the brain and resemble a puff of smoke.

Besides being a rare condition, epidemiological studies showed that MMD is most prevalent in East Asian countries with an estimated prevalence of 3.2–10.5 per 1,000,000 and estimated annual incidence of 0.43–0.94 per 100,000 [2]. The incidence and prevalence of MMD are increasing with recent advances in neuroradiological diagnostic modalities. In the current literature, there are no precise data on the incidence of MMD in Europe and the United States. Most recent US study estimated overall incidence as 0.086 per 100,000 [3]. Between Western countries, MMD is exceptionally rare in African Americans; it presumably remains a misdiagnosed cause of ischemic and/or hemorrhagic stroke. MMD has two age peaks: in childhood and in young adults. Worldwide studies showed women are more frequently affected than men [4].

Although several hypotheses have been investigated, the pathogenesis of this enigmatic disease remains obscure. Here, we report the case of MMD in a 29-year-old African American female who presented with an ischemic stroke for the second time that manifested after pregnancy. This case is important to increase awareness of the probability of this rare disease in Western countries as well as to call attention to pregnancy's accelerating effects of MMD. Pregnancy and puerperium have some potential deleterious effect on MMD, but mechanisms remain unclear [5].

## 2. Case Description

**Figure 1:**
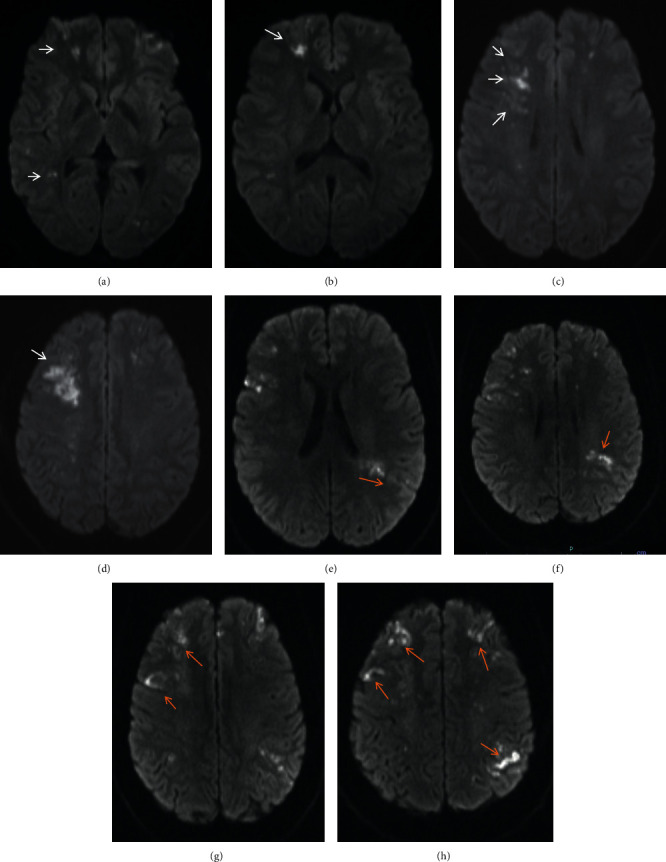
Axial MRI brain imaging: (a)—(d) images from the first episode of stroke, with regions of restricted diffusion indicative of acute ischemia (arrows), primarily noted in the right cerebral hemisphere; (e)—(h) images from the second episode, with progressive bilateral ischemic changes (arrows).

**Figure 2:**
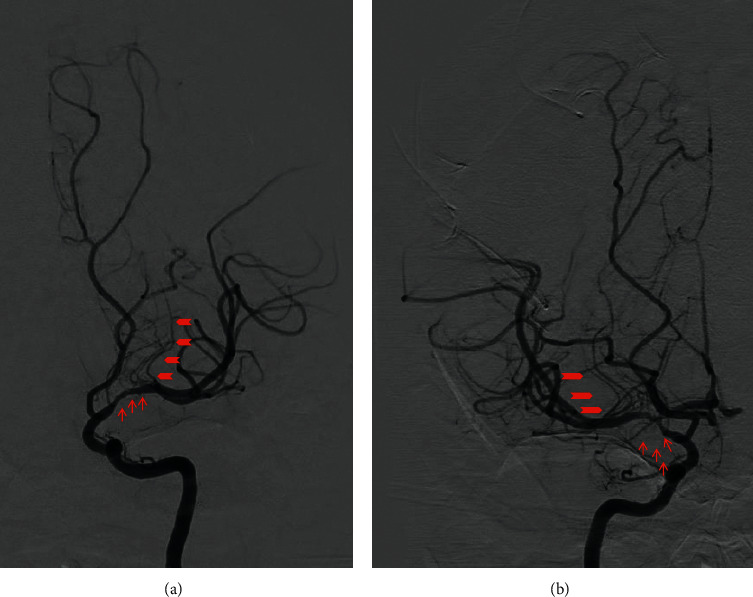
Digital subtraction angiography images: (a) left internal cerebral artery (ICA) with focal distal ICA and proximal M1 arteriopathy (arrows) with prominent lateral lenticulostriate branches, indicating early collateralization of flow (arrowheads); (b) right internal cerebral artery (ICA) with focal distal ICA, proximal MCA (M1), and proximal ACA (A1) arteriopathy (arrows) with prominent lateral lenticulostriate branches, indicating early collateralization of flow (arrowheads).

The patient is a 29-year-old African American female, G1P1A0, with a history of stroke in multiple areas in the brain approximately two months ago; she was eight months postpartum following an uneventful pregnancy and normal vaginal delivery. At this time, the patient presented with confusion, unusual behavior pattern, headache, involuntary movements, and forgetfulness for the last three days. Patient reported that she had difficulty in performing easy tasks such as putting a bottle together, getting out of the car, and changing her baby. She also reported that, at times, she got lost in her home. She denied having weakness, numbness, seizures, chest pain, shortness of breath, fever, or chills. The patient had a previous ischemic stroke affecting her left side but no residual weakness at this time; however, she did report urinary incontinence since then. Multiple members in her family had a stroke when they were young. The patient was awake, alert, and oriented with no focal neurologic deficits. National Institutes of Health Stroke Scale (NIHSS) score was zero. The first stroke was two months ago, and at that time, she presented with left-sided weakness and cognitive deficits. She was found to have acute cerebral ischemic infarcts primarily noted in the right cerebral hemisphere (Figures 1(a)–1(d)). Subsequently, she underwent a CT angiogram (CTA), which showed questionable stenosis of the bilateral middle cerebral artery (MCA) and distal right vertebral artery. On this admission, she had further progression of intracranial stenosis and ischemic infarcts noted bilaterally (Figures 1(e)–1(h)). CTA of the head revealed moderate to severe narrowing of the supraclinoid segment of both internal carotid arteries (ICA) and extensive narrowing of both M1 segments of the MCAs and both A1 segments of anterior cerebral arteries (ACA). Cerebral perfusion imaging revealed diminished cerebral blood flow in the ACA, MCA, and watershed territories, compatible with ischemia. A diagnostic digital subtraction cerebral angiography showed progressive arteriopathy in both ICA terminals, M1, and A1 with prominent lateral lenticulostriate branches, indicating early collateralization of flow (Figures 2(a) and 2(b)). Based on these clinical and radiological findings, our diagnostic considerations were MMD, primary CNS vasculitis, hypertensive disorder preceding multiple strokes, or hypercoagulability disorders. During this admission, her blood pressure was well controlled. There were no skipped lesions found to support vasculitidies. She denied the previous history of photosensitivity and rash on the face or limbs. There was no history of oral or nasal ulcers, hemoptysis, hematemesis, and recurrent urine infections. She denied a history of Raynaud phenomenon, and there was no history of blood clots that she is aware of. Detailed history and review of systems did not elicit any suspicion of rheumatologic disease. Basic blood work was within normal limits. Specific tests for hypercoagulability, antiphospholipid syndrome, and rheumatologic disorders were negative. CSF analysis was also unremarkable. In following with diagnostic criteria, we diagnosed our patient with MMD. We discharged patients with antihypertensive medications, high-dose statin, and dual antiplatelet therapy.

## 3. Discussion

MMD is an important diagnosis to consider when evaluating cerebrovascular events in children, young adults, and Asian ethnicity. Cerebrovascular events are the primary presenting symptoms and are related both to the occlusion of the ICAs and their main branches. The incidence of MMD in Europe and the United States is not well studied, but literature analysis suggests that the incidence in Western countries is lower than in Asian countries [3, 6]. Clinical and demographic presentation of MMD cases is quite different between ethnicities. The results of the US-based studies suggest that Caucasian patients are characterized by an older average age of presentation and without bimodal age distribution [7]. Comparing to Asian cases, Caucasian patients mostly present with ischemic strokes with more benign symptoms and less common familial occurrence [4, 8, 9]. MMD is especially rare in African American ethnicity. Uchino et al. [3] detected only 27 MMD cases in African American race, 13 of whom were diagnosed with moyamoya syndrome with having concurrent sickle cell disease.

The diagnosis of MMD requires detection of steno-occlusive changes in the ICA either by CTA, MRA, or cerebral angiography. In patients with unilateral stenosis, cerebral angiography is needed for definitive diagnosis, while bilateral cases can be promptly diagnosed with either CTA or MRA. Cerebral angiography is more sensitive than CTA to show small saccular aneurysms and collateral moyamoya vessels that is why cerebral angiography is considered the gold standard in diagnosis of MMD [10]. Angiographic staging of the MMD was described by Suzuki and Takaku [11] to represent the natural course of the angioarchitecture in affected patients. Although Suzuki's angiographic staging explains compensatory mechanisms of ischemic changes, it is not able to reflect the severity of the MMD. Recently, Czabanka et al. [12] described more specific grading criteria which allow to stratify angiographic severity and clinical symptomatology in MMD patients. There are no remarkable specific CSF changes in patients with MMD. Electroencephalography (EEG) appearances can be abnormal but are usually nonspecific. Infectious processes and primary central nervous system vasculitis should be in the main differential diagnosis. All rheumatologic workups were negative including CSF analysis.

Currently, the pathophysiological mechanisms of MMD are mainly unknown. Investigators described genetic factors and inflammatory changes as the two most representative mechanisms. Recent genetic studies pointed RNF213 as an important susceptibility gene of MMD. A big study on zebrafish vasculature showed that RNF213 is involved in a novel signaling pathway in intracranial angiogenesis [13]. Further studies are needed to explain how the mechanisms of RNF213 mutations are leading to clinical features of MMD. In addition to underlying genetic factors, angiogenesis and vasculogenesis-related inflammatory and autoimmune factors seem to be contributing to the pathophysiology of MMD. Masuda et al. [14] detected intimal hyperplasia of brain vasculature caused by macrophages and T lymphocytes. In light of the current studies, one single mechanism is unable to explain MMD pathogenesis, and further studies are needed.

Unfortunately, there is no definitive treatment described in the literature shown to reverse the intracranial vasculopathy of MMD. Due to the progressive nature of MMD, the prognosis is poor. Current studies show that, within the first year of diagnosis of MMD, the risk of recurrent stroke was approximately 18% but decreased to 5% per year henceforth [7, 15]. Currently, treatment strategies are focused on treating symptoms and halting progression of arteriopathy. The guidelines recommend the use of antiplatelet agents in acute and symptomatic chronic phase patients except in those who present with hemorrhage [16]. The surgical indications and timing of revascularization for MMD have not been established well. Revascularization surgery aims toward improving brain perfusion to prevent recurrent strokes. Due to a wide variety of disease presentation, the decision of revascularization surgery should be specific to each individual's condition. In our case, due to satisfactory response to initial medical management, we did not consider surgical intervention.

## 4. Conclusion

MMD is a multifactorial, complex, and progressive cerebrovascular occlusive disease that has mainly unknown etiology. Since the diagnosis of MMD is rapidly increasing worldwide, we highly suggest considering MMD as a differential diagnosis in Western countries, especially in young patients who present with progressive cerebrovascular events. Long-term close follow-up is crucial to minimize the risk of future cerebrovascular events and improve outcomes.
